# Capillary Flow-Based One-Minute Quantification of Amyloid Proteolysis

**DOI:** 10.3390/bios14080400

**Published:** 2024-08-19

**Authors:** Taeha Lee, Da Yeon Cheong, Kang Hyun Lee, Jae Hyun You, Jinsung Park, Gyudo Lee

**Affiliations:** 1 Department of Biotechnology and Bioinformatics, Korea University, Sejong 30019, Republic of Korea; xogk0038@korea.ac.kr (T.L.); 2017270450@korea.ac.kr (D.Y.C.); leoigu97@korea.ac.kr (K.H.L.); 2 Interdisciplinary Graduate Program for Artificial Intelligence Smart Convergence Technology, Korea University, Sejong 30019, Republic of Korea; 3 Department of Digital Management, Korea University, Sejong 30019, Republic of Korea; hyuni22@korea.ac.kr; 4 Department of Biomechatronic Engineering, College of Biotechnology and Bioengineering, Sungkyunkwan University, Suwon 16419, Republic of Korea; 5 Department of MetaBioHealth, Sungkyunkwan University, Suwon 16419, Republic of Korea; 6 Department of Biopharmaceutical Convergence, Sungkyunkwan University, Suwon 16419, Republic of Korea

**Keywords:** capillary, amyloid, lab on paper, hen-egg-white lysozyme, trypsin, autolysis

## Abstract

Quantifying the formation and decomposition of amyloid is a crucial issue in the development of new drugs and therapies for treating amyloidosis. The current technologies for grasping amyloid formation and decomposition include fluorescence analysis using thioflavin-T, secondary structure analysis using circular dichroism, and image analysis using atomic force microscopy or transmission electron microscopy. These technologies typically require spectroscopic devices or expensive nanoscale imaging equipment and involve lengthy analysis, which limits the rapid screening of amyloid-degrading drugs. In this study, we introduce a technology for rapidly assessing amyloid decomposition using capillary flow-based paper (CFP). Amyloid solutions exhibit gel-like physical properties due to insoluble denatured polymers, resulting in a shorter flow distance on CFP compared to pure water. Experimental conditions were established to consistently control the flow distance based on a hen-egg-white lysozyme amyloid solution. It was confirmed that as amyloid is decomposed by trypsin, the flow distance increases on the CFP. Our method is highly useful for detecting changes in the gel properties of amyloid solutions within a minute, and we anticipate its use in the rapid, large-scale screening of anti-amyloid agents in the future.

## 1. Introduction

The accumulation of amyloids has been extensively researched in the field of medical science for many years, especially in connection with degenerative disorders such as dialysis-related amyloidosis, AL amyloidosis, and Alzheimer’s disease [1,2,3,4,5]. These accumulated amyloids, specifically insoluble fibrous aggregates, play a pivotal role in causing damage and functional disabilities in tissues and organs [6,7,8]. Therefore, understanding the formation and degradation mechanisms of these proteins is a significant concern in biochemical and medical science [9,10,11,12]. Comprehensive insights into these proteins have been attained through traditional methods like fluorescence analysis using thioflavin-T, secondary structure analysis via circular dichroism, and advanced imaging techniques such as atomic force microscopy and transmission electron microscopy [13,14,15,16]. However, these methods, which require sophisticated spectroscopy or nano-scale imaging equipment and involve considerable analysis time, limit the rapid screening of potential amyloid-degrading drugs.

To addresses these limitations, paper-based microfluidics, or microfluidic paper-based analytical devices (µPADs), have emerged as a versatile and cost-effective platform for various biochemical assays [17,18,19]. Capillary flow-based paper (CFP), a subtype of µPADs, has been introduced as a promising alternative [20,21,22]. This makes them particularly suitable for point-of-care testing and field diagnostics, where simplicity and portability are crucial. 

The simplicity and versatility of CFP have been demonstrated in a wide range of applications. For example, Xia et al. developed a CFP material to detect lipase, which is the biomarker for acute pancreatitis [22]. Zhao et al. proposed a CFP-based method for the detection of lipase, which is associated with inflammation, oncogenesis, and allergic reactions [23]. Liu et al. utilized CFP to sense both chymotrypsin and its inhibitors [24]. All of these studies are based on the viscosity changes resulting from alginate, polysaccharide, and gelatin, respectively [22,23,24]. In detail, the viscosity of a solution inherently can dictate its resistance to capillary flow on CFP [25]. Similar to alginate, polysaccharide, and gelatin, amyloid also has distinct gel-like physical properties as an insoluble denatured polypeptide [26,27,28]. This similarity suggests that amyloid degradation can be investigated using paper-based microfluidics by monitoring viscosity changes, but this has not been reported so far.

In this study, we investigate the performance of CFP in quantifying amyloid concentration and its proteolysis. Using a hen-egg-white lysozyme amyloid solution as a baseline model, experimental conditions were calibrated to achieve consistent flow-distance measurements. The gel-like physical properties of the amyloid solution result in shorter flow-paths on CFP compared to those in pure water. We observed a significant increase in the flow distance corresponding to amyloid degradation by trypsin. We believe this novel approach holds immense potential and could redefine amyloid analysis methodologies. Additionally, it could significantly accelerate large-scale drug-screening processes, laying the groundwork for future therapeutic innovations.

## 2. Materials and Methods

### 2.1. Materials

Hen-egg-white lysozyme (HEWL), trypsin from porcine pancreas, and thioflavin-T (ThT) were sourced from Sigma-Aldrich (St. Louis, MO, USA). Hydrochloric acid (HCl) and sodium hydroxide (NaOH) were obtained from DAEJUNG Chemical & Metals (Seoul, Republic of Korea). Distilled water (DW) and phosphate-buffered saline (PBS, pH 7.4, 1×) were obtained from Gibco (West Norriton, PA, USA). Universal indicator paper was acquired from Doosan Scientific (Seoul, Republic of Korea). A membrane syringe filter (0.2 μm, Whatman GD/X 25) was procured from Cytiva (Marlborough, MA, USA). Polypropylene (PP) film was purchased from Webis (Seoul, Republic of Korea).

### 2.2. Preparation of HEWL Amyloid

For the formation of HEWL amyloid, HEWL was prepared at a concentration of 10 wt% in DW that had been adjusted to pH 2 using a 1 M HCl solution [29]. This solution was then filtered through a membrane syringe filter (pore size: 0.2 μm). After filtration, the HEWL solution was transferred into a sterilized vial, sealed, and incubated in an oil bath for 3 days. The resulting HEWL amyloid was neutralized to pH 8 using a 1 M NaOH solution and subsequently diluted with DW (pH 8) to produce amyloids at various concentrations. 

### 2.3. Characterization of the HEWL Amyloid Fibrils

The morphology of the HEWL amyloid fibril was obtained using atomic force microscopy (AFM) (MultiMode VIII, Bruker, Billerica, MA, USA) [30]. For AFM sample preparation, 20 μL aliquots of the sample were deposited onto freshly cleaved mica, incubated for 5 min, rinsed with 200 μL of DW, and dried with N_2_ gas. ThT assays were conducted using a hybrid multimode reader (Synergy H1, Biotek, Oviedo, FL, USA) at an excitation wavelength of 440 nm and an emission wavelength of 485 nm [31]. The ThT concentration of the reaction solution was adjusted to 20 μM.

### 2.4. Trypsin Treatment of HEWL Amyloids

**Figure 1:**
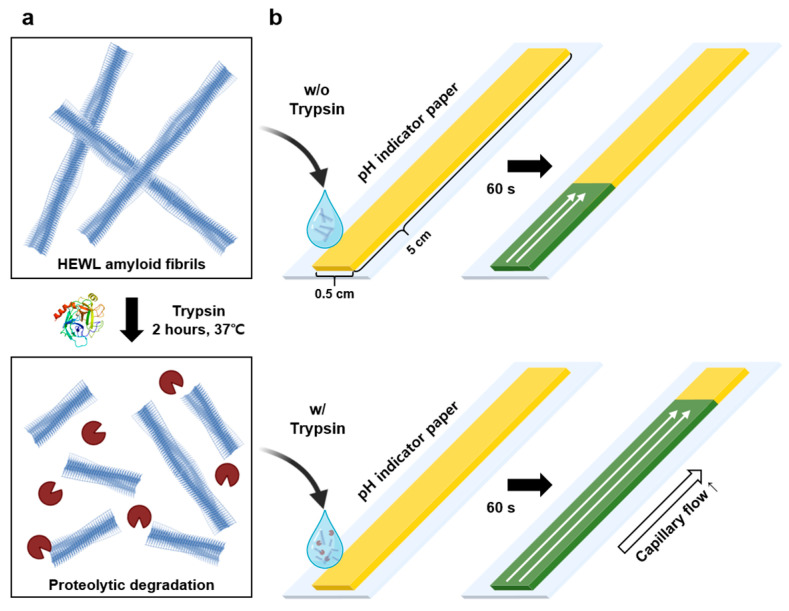
Schematic of (**a**) HEWL amyloid and its proteolytic degradation by trypsin and (**b**) comparative analysis of the capillary flow of amyloid solution on CFP treated and untreated with trypsin.

For the proteolytic degradation of HEWL amyloids, trypsin was directly dissolved in the HEWL amyloid solution (Figure 1a). After ensuring homogeneity through vortexing, the mixture was incubated at 37 °C, with mixing performed at 20-min intervals over 2 h. For negative control experiments, thermally denatured trypsin was prepared at 60 °C for 24 h [32].

### 2.5. Fabrication of the CFP

To fabricate CFP, we employed universal pH indicator paper, which is commercially designed for fluid absorption and movement (Figure 1b). The pH indicator paper was trimmed to dimensions of 50 mm × 5 mm (length × width) using a cutting machine. These pieces of pH indicator paper were then affixed to PP film with double-sided tape to ensure precision during experimental procedures. We fabricated an optimal piece of CFP by attaching four pH indicators to a PP film (70 mm × 60 mm), and its optimality was determined using high-resolution images taken with a smartphone camera. We employed a multi-channel pipette (Eppendorf, Hamburg, Germany) to dispense solutions simultaneously onto the CFP.

### 2.6. Theoretical Background and Data Analysis

Conventionally, capillary action is characterized using the Lucas–Washburn (LW) model (Equation (1)) [33].

(1) $$D_{F}\left(t\right)=\sqrt{\frac{\gamma r\cos\theta }{2\mu }t}$$

Within this formulation, flow distance (*D_F_*) over time describes the progression of a solvent front driven by capillary pressure, and *γ* symbolizes the surface tension between the liquid and air interfaces. *r* corresponds to the effective radius of the capillary channel, and *θ* indicates the contact angle between the liquid phase and the channel. The liquid’s viscosity is represented by *μ*, and *t* designates the elapsed time. However, the LW equation, originally developed for cylindrical capillaries, assumes a consistent and uninterrupted flow driven purely by capillary forces without accounting for evaporation. In practical applications involving paper-based microfluidics, fluid loss due to evaporation plays a significant role, especially over extended periods and in open systems [34,35]. The porous and hydrophilic nature of paper significantly impacts the corresponding fluid dynamics, wherein evaporation cannot be neglected. To address these limitations and provide a more accurate description of capillary action in paper-based systems, we used a new expression derived from Darcy’s law [36]. This approach allows us to incorporate the effects of evaporation and the unique structural properties of paper, offering a more comprehensive model for fluid movement in these systems. The derived equation is as follows:

(2) $$D_{F}\left(t\right)=\sqrt{\frac{\gamma r\phi h\cos\theta }{4\mu q_{0}}\left(1-e^{-\frac{2q_{0}t}{\phi h}}\right)}$$

where *ϕ* is the porosity of the paper, *h* is the thickness of the porous channel, and *q*_0_ is the evaporation rate defined as the volume of liquid evaporated per unit area and time from the wetted channel. At the limit, that is, the point where there is no evaporation in the channel (*q*_0_ ⟶ 0), this equation reduces to the LW equation.

To achieve a precise quantification of fluid movement on CFP, we captured CFP images using a smartphone (Galaxy S22, Samsung, Suwon-si, Republic of Korea). The *D_F_* was subsequently analyzed using ImageJ software (version 1.54d, NIH). To compute the *D_F_*, the ‘solvent front’ region pixel value (*P_sf_*) of the CFP was divided by the total area pixel value (*P_total_*), and this ratio was then multiplied by the CFP’s length (*L_CFP_*, 50 mm), as shown below.

*D_F_* = *P_sf_*/*P_total_* × *L_CFP_* (3)

Through Equation (2), it can be discerned that *D_F_* is inversely proportional to *μ*. Furthermore, there is direct proportionality between the concentration of trypsin and *D_F_*. Hence, the relationship between *D_F_*, *μ*, and trypsin concentration can be elucidated as follows:

(4) $$D_{F}\propto \frac{1}{\sqrt{\mu }}\propto \left[Trypsin\right]$$

Based on this theoretical background, we predict that as the concentration of trypsin increases, there is subsequent amyloid degradation, leading to a reduction in the viscosity of the amyloid solution, which, in turn, elevates the *D_F_*.

## 3. Results

### 3.1. Morphological and Fluorescent Analysis of HEWL Amyloid Degradation

**Figure 2:**
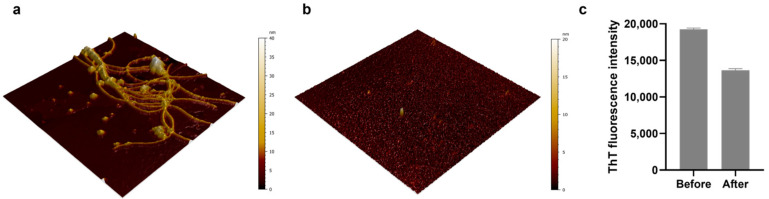
AFM images (5 × 5 μm^2^) of (**a**) HEWL amyloid fibrils and (**b**) trypsin-treated amyloid sample. (**c**) ThT fluorescence intensity measurements taken before and after proteolytic degradation by trypsin. The data were obtained through triplicate measurements.

Previous studies have shown that proteases capable of protein hydrolysis, such as pepsin and trypsin, are effective in degrading amyloid [30,37,38]. Notably, trypsin is known to be able to degrade lysozyme amyloids, which are associated with hereditary lysozyme amyloidosis [37]. Given this background, we utilized AFM and fluorescence analysis to examine the potential degradation of HEWL amyloid by trypsin. In the AFM images, we observed HEWL amyloid fibrils (Figure 2a) and an amyloid fragment formed via trypsin-induced hydrolysis (Figure 2b). As shown in Figure 2a, the AFM images displayed multiple strands of HEWL amyloid fibrils. This amyloid morphology is consistent with AFM images in previous reports [29,30,39,40,41]. In contrast, the images of the trypsin-treated samples displayed only nanoscale fragments, indicative of degraded amyloids. Along with the morphological comparison of the amyloid samples before and after trypsin treatment, we conducted ThT fluorescence assays to investigate the β-sheet structures within the amyloids. As a result, the ThT fluorescence intensity of the bare amyloid and the trypsin-treated amyloid samples decreased from 19,265 ± 130 (mean ± standard deviation) to 13,639 ± 191 (Figure 2c). These findings confirm that trypsin can degrade HEWL amyloids.

### 3.2. Development of the CFP Analytical Platform

**Figure 3:**
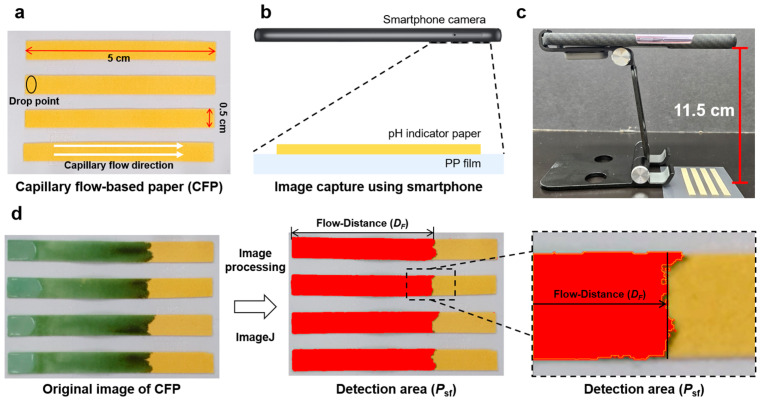
(**a**) CFP fabricated by attaching four pH indicators to a PP film. Solutions were dispensed at the drop point on the CFP. (**b**) Schematic illustration and (**c**) side view of a smartphone mounted on a stand, capturing a piece of CFP from a distance of 11.5 cm. (**d**) Image-processing procedure used for quantifying the detection area (*P*_sf_) on the CFP to accurately calculate the detection factor (*D_F_*).

To effectively evaluate the *D_F_* of the CFP, we designed it such that simultaneous measurement of up to four samples could be performed (Figure 3a). By adopting a systematic approach, we aimed to enhance the accuracy and efficiency of the measurements. Upon completion of the setup, we then proceeded to capture high-resolution images of the pieces of CFP using a readily available smartphone (H.264, AAC, 30 fps), as shown in Figure 3b,c. The smartphone was mounted on a smartphone holder, with the distance between the smartphone and the CFP fixed at 11.5 cm. For subsequent analysis of these images, we used ImageJ software (version 1.54d), a versatile tool known for its robust image-processing capabilities. Specifically, we utilized two plugins, ‘color threshold’ and ‘particle analysis’, which enabled us to differentiate and identify the solvent front area (*P*_sf_) as a detection area based on color intensities. We extracted the *P*_sf_, which is distinctly represented as the red region in the original images, and subsequently calculated the *D_F_* using Equation (3), as depicted in Figure 3d.

### 3.3. CFP Performance for HEWL Amyloid Concentration

**Figure 4:**
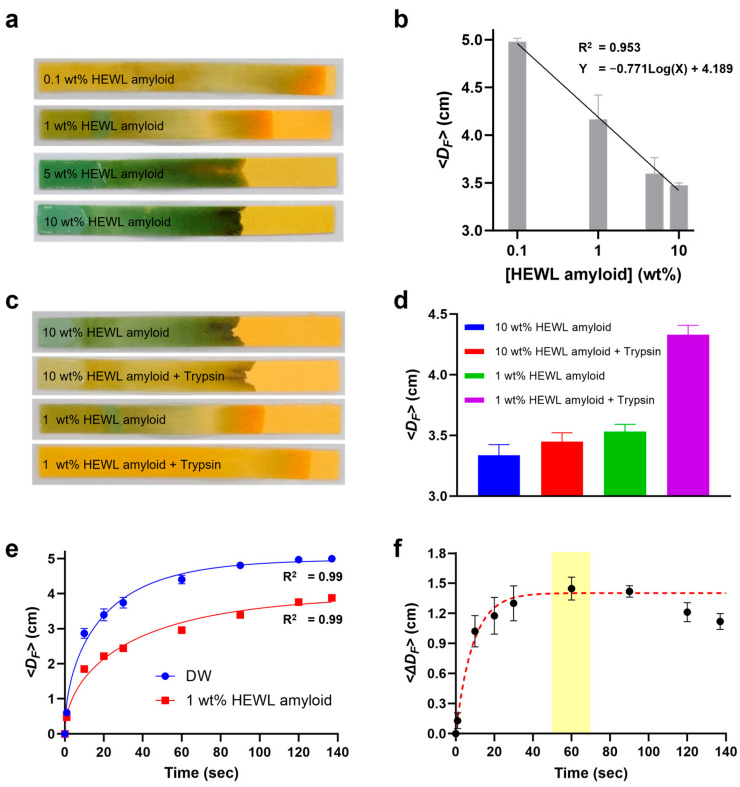
Optimization of the CFP for amyloid solution. (**a**) Photo of CFP and (**b**) *D_F_* of different concentrations of HEWL amyloids. (**c**) Photo of CFP and (**d**) the *D_F_* between trypsin-treated and untreated HEWL amyloids. (**e**) Kinetics analysis of 1 wt% HEWL amyloid and DW based on a Darcy-based modified LW model, wherein *D_F_*(*t*) = $\sqrt{a\left(1-e^{bt}\right)}$. (**f**) A dynamic comparative analysis illustrating the difference in *D_F_* (Δ*D_F_*) between the kinetic curves of DW and 1 wt% amyloid at each timepoint, with the plateau timepoint (60 s) highlighted in yellow.

To investigate the performance of CFP with respect to amyloid solution, we analyzed the *D_F_* for various concentrations of HEWL amyloid solutions ranging from 0.1 to 10 wt%. Specifically, HEWL solutions (50 μL) were dispensed onto the CFP using a multi-channel pipette. After a duration of 180 s, an image of the CFP was captured, showing that the *D_F_* decreased as the amyloid concentration increased (Figure 4a). This phenomenon can be attributed to the nature of amyloids as insoluble denatured polymers. In detail, as the amyloid concentration increases, interactions between amyloid fibrils become more prominent, as reflected by a gel-like property [42]. At a certain elevated concentration (5 wt%), the solution of amyloid undergoes a phase transition, shifting from a sol state to a gel state. Meanwhile, the CFP utilized in the experiment turn green at pH values above 8, light green at pH 7, and orange at pH values below 6 [22]. This pH indicator paper not only measures solution pH changes but also provides information about *D_F_*, offering an advantage over traditional colorimetric paper- or hydrogel-based sensors [43,44,45]. As shown in Figure 4a, a concentration-dependent decrease in pH was observed as the HEWL amyloid was diluted with DW.

When plotting *D_F_* against amyloid concentration, an inverse relationship with good linearity (R^2^ = 0.953) on a logarithmic scale was evident (Figure 4b). The *D_F_* of the 0.1 wt% HEWL amyloid solution almost reached the endpoint of CFP, meaning that the viscosity of the solution was at the DW level. Although the 5 wt% amyloid solution was diluted by half with respect to the original amyloid solution (10 wt%), the *D_F_* between them showed little difference. Consequently, we conducted subsequent experiments using both low-concentration (1 wt%) and high-concentration (10 wt%) HEWL amyloid solutions.

### 3.4. CFP Test for Trypsin-Treated HEWL Amyloid

CFP tests were performed on the trypsin-treated amyloid solutions, and the *D_F_* values were compared. The HEWL amyloid solutions (1 and 10 wt%) were treated with trypsin (8 mg/mL) and incubated at 37 °C for 2 h. Both the trypsin-treated and untreated amyloid solutions (50 μL) were simultaneously dispensed onto the CFP, and a CFP image was captured after 120 s (Figure 4c). Given the larger gel-to-sol transition, we hypothesized that there would be a more pronounced *D_F_* difference in the 10 wt% amyloid solution after trypsin treatment compared to the 1 wt% solution. However, interestingly, the experimental results (Figure 4d) showed that the *D_F_* difference as a percentile was more noticeable in the 1 wt% amyloid solution (22%) than in the 10 wt% solution (6%). It can be inferred that the 10 wt% amyloid solution maintained a gel-to-gel state, albeit of proteolysis, resulting in minor *D_F_* changes [28]. This is consistent with the negligible difference in *D_F_* observed between the 5 wt% and 10 wt% amyloid solutions, as depicted in Figure 4b. By contrast, the 1 wt% amyloid solution underwent a significant transition from gel to sol [28], resulting in a pronounced change in *D_F_*.

After trypsin treatment, the 10 wt% amyloid solution exhibited a more significant pH drop than the 1 wt% solution (Figure 4c), suggesting enhanced amyloid proteolysis. During proteolysis, the carboxyl groups of amino acids become more numerous owing to peptide bond breakdown, leading to a rise in dissociated hydrogen ions and a subsequent reduction in solution pH [46,47]. Our result implies that while trypsin digested more amyloid in the 10 wt% solution, no gel-to-sol phase transition happened. This phenomenon underscores the importance of amyloid density when evaluating amyloid proteolysis using CFP. Thus, we adopted 1 wt% HEWL amyloid solution as an optimal condition for further experiments with CFP.

### 3.5. CFP Kinetics for Optimization of Analysis Time

To delve deeper into comprehending the capillary action of amyloid solutions in CFP, we embarked on a series of kinetics analyses. We used a 1 wt% amyloid solution for these analyses, and hypothesizing that there would be complete degradation of amyloid, we employed DW as a negative control for a comparative study (Figure 4e). The *D_F_* for both solutions increased over time, and DW exhibited a higher *D_F_* rate than the amyloid solution, as expected. A significant observation was the steep initial increase in the kinetics curves for both fluids, transitioning to a more gradual ascent post 10 s. This phenomenon is attributed to capillary action, as the solution is rapidly drawn into the nano-porous structure of CFP and gradually transitions to a steady state [48]. Our results do not conform to the conventional LW model but rather match better with the modified LW model using Darcy’s law. The data were fit to the following equation:

(5) $$D_{F}\left(t\right)=\sqrt{a\left(1-e^{bt}\right)}$$

The parameter *a* is given by the following equation:

(6) $$a=\frac{\gamma r\phi hcos\theta }{4\mu q_{0}}$$

The parameter *b* is expressed as follows:

(7) $$b=\frac{2q_{0}}{\phi h}$$

We found that the optimal fitting values for DW are *a* = 24.73 and *b* = 0.03, while for HEWL, the optimal fitting values are *a* = 15.58 and *b* = 0.02. This equation (Equation (5)) models the growth pattern of the *D_F_*; here, *a* represents the asymptotic maximum value of *D_F_*, and *b* represents the rate at which the *D_F_* approaches its maximum. The use of this model allows for capturing the nonlinear dynamics observed in the experiments more accurately. Calculating *q*_0_ based on *a* could lead to inaccuracies due to the potential variability in the asymptotic maximum values, which may be influenced by external factors. Therefore, we used the parameter *b* to calculate *q*_0_ as it directly reflects the evaporation rate. Although there are no exact studies analyzing the porosity and height of the CFP we used, the porosity (*ϕ*) of similar filters is known to be approximately 0.6, and the thickness (*h*) is approximately 220 μm [49]. Based on this, we calculated the evaporation rates (*q*_0_) as follows: for DW, *q*_0_ ≈ 1.94 μm/s, and for HEWL, *q*_0_ ≈ 1.09 μm/s. These values indicate that DW has a higher evaporation rate compared to the amyloid solution, which aligns with our observations of the *D_F_* rates. This suggests that the higher viscosity and gel-like properties of the amyloid solution result in a slower evaporation rate, thus impacting the capillary flow dynamics in the CFP system. Additionally, by applying the modified LW equation, the fit accuracy for the *D_F_* of the DW and HEWL improved significantly (R^2^ = 0.99).

The new model derived from Darcy’s law provided more accurate predictions by accounting for fluid loss due to evaporation. This model effectively explained the nonlinear kinetics observed in the experiments, particularly reflecting the characteristic transition from an initial steep increase to a gradual one. This study demonstrates that the modified model applying Darcy’s law can more precisely describe capillary action in the CFP system, suggesting that this approach could be useful for analyzing various biological materials.

To determine the optimal analysis time for CFP, a dynamic comparative analysis was conducted by calculating Δ*D_F_* through the subtraction of the two kinetic curves presented in Figure 4e. The Δ*D_F_* continued to increase for 30 s and then reached saturation; then, it gradually decreased after 60 s (Figure 4f). This behavior is due to the potential energy of capillary action decreasing as the solution moves toward the CFP’s endpoint (50 mm). Excluding two data points post 100 s and applying an exponential fit yielded a robust correlation (R^2^ = 0.96). We confirmed that the minimum time point at which the plateau becomes apparent is 60 s, and, consequently, we have determined that this time-point is the optimal analysis time for conducting amyloid experiments based on CFP.

### 3.6. CFP-Based Quantification of Amyloid Degradation

**Figure 5:**
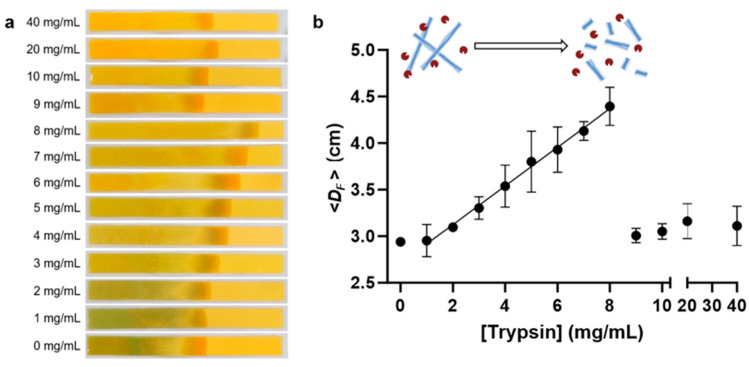
CFP-based quantification of amyloid proteolysis by trypsin. (**a**) Photo of CFP and (**b**) the calculated *D_F_* against trypsin concentration (0 to 40 mg/mL). The data were obtained through triplicate measurements (mean ± standard deviation).

We investigated the quantification of amyloid degradation by trypsin using CFP, employing the optimal conditions determined in previous experiments: HEWL amyloid concentration (1 wt%) and analysis time (60 s). Amyloid proteolysis was tested across 12 different trypsin concentration conditions ranging from 1 to 40 mg/mL, and the *D_F_* value was quantified through CFP analysis. Figure 5a indicates that as the trypsin concentration increased in 1 mg/mL intervals, a gradual expansion in the solvent-front was observed. Notably, CFP seems to show poor performance at high trypsin concentrations (>8 mg/mL). To analyze this phenomenon in detail, the *D_F_* was calculated and plotted against trypsin concentration (Figure 5b). *D_F_* increased proportionally with trypsin concentration, with a pronounced linearity observed within the concentration range of 1 to 8 mg/mL. Through an analysis of the proteolysis effect of trypsin on a 1% HEWL amyloid solution (pH 8 and 37 °C), the LOD and sensitivity values were determined to be 2 mg/mL and 2.07 mm·L/g, respectively. This indicates that the extent of amyloid hydrolysis by trypsin can be precisely analyzed using CFP. However, beyond a certain trypsin concentration (>8 mg/mL), a drastic decline of approximately 35% in the *D_F_* was observed. This phenomenon is attributed to trypsin autolysis or potential distortion of the protein molecule due to high trypsin concentrations [50], and it does not reflect an issue with CFP itself. These findings suggest that CFP not only facilitates easy analysis of amyloid proteolysis but also enables the determination of the appropriate protease concentration for analysis.

**Figure 6:**
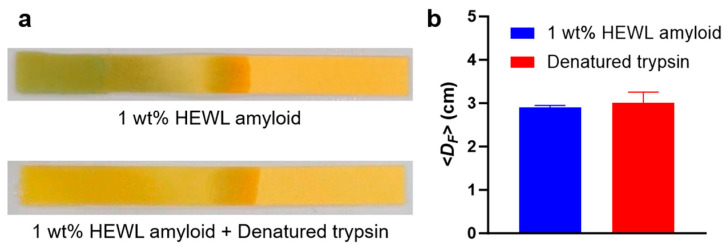
(**a**) Photo of CFP and (**b**) the calculated *D_F_* for HEWL amyloids with and without inactivated trypsin. The data were obtained through triplicate measurements (mean ± standard deviation).

To validate whether the degradation of HEWL amyloid was genuinely due to the activity of trypsin, we examined the hydrolysis of amyloid by thermally denatured trypsin. To ensure robust experiments, we adopted a trypsin concentration of 8 mg/mL, which appeared to induce the highest amyloid degradation under our experimental conditions. As shown in Figure 6, the denatured trypsin did not induce amyloid degradation at all compared to the negative control (amyloid only). As a result, it was concluded that the observed *D_F_* change in CFP could indeed be attributed to amyloid degradation by trypsin.

## 4. Conclusions

In this study, we introduced capillary flow-based paper (CFP) as a fast and efficient method for quantifying amyloid degradation, addressing the challenges associated with existing techniques that require complex equipment and extensive analysis times. The amyloid solution exhibits gel-like properties, leading to a distinctive flow-distance (*D_F_*) pattern within CFP. Through the analysis of *D_F_* concerning amyloid concentration, the investigation of *D_F_* and pH changes resulting from amyloid hydrolysis, and kinetic analyses, we determined the optimal amyloid concentration and analysis time for CFP experiments. We demonstrated that CFP can be utilized to analyze the extent of protease-induced amyloid hydrolysis. Furthermore, we confirmed that CFP is an effective tool for exploring the protease concentration range that yields the best performance in amyloid hydrolysis. Our CFP-based analytical method will facilitate the screening for amyloid inhibitors or degraders and suggest an optimal concentration range. We emphasize the potential of CFP as an excellent alternative technology capable of saving both time and resources in amyloid research. While our current study focused on purified amyloids, future work will explore the application of CFP to various biological fluids to enhance its accuracy and applicability in amyloid drug-screening evaluations.

## Data Availability

The original contributions presented in the study are included in the article, further inquiries can be directed to the corresponding authors.
